# Some Key Factors Influencing the Flame Retardancy of EDA-DOPO Containing Flexible Polyurethane Foams

**DOI:** 10.3390/polym10101115

**Published:** 2018-10-09

**Authors:** Agnieszka Przystas, Milijana Jovic, Khalifah A. Salmeia, Daniel Rentsch, Laurent Ferry, Henri Mispreuve, Heribert Perler, Sabyasachi Gaan

**Affiliations:** 1 Additives and Chemistry, Advanced Fibers, Empa Swiss Federal Laboratories for Materials Science and Technology, Lerchenfeldstrasse 5, 9014 St. Gallen, Switzerland; przystas.agnieszka@empa.ch (A.P.); milijana.jovic@empa.ch (M.J.); khalifah.salmeia@empa.ch (K.A.S.); 2 Laboratory for Functional Polymers, Empa Swiss Federal Laboratories for Materials Science and Technology, Überlandstrasse 129, 8600 Dübendorf, Switzerland; daniel.rentsch@empa.ch; 3 Centre des Matériaux des Mines d’Alès (C2MA), IMT Mines Ales, Université de Montpellier, 6 Avenue de Clavières, F-30319 Alès Cedex, France; Laurent.Ferry@mines-ales.fr; 4 FoamPartner Fritz Nauer AG, 8633 Wolfhausen, Switzerland; Henri.Mispreuve@foampartner.com (H.M.) Heribert.Perler@foampartner.com (H.P.)

**Keywords:** flexible polyurethane foam, flame retardant, bridged–DOPO compounds, microscale combustion analysis, thermal analysis

## Abstract

The role of various additives (emulsifier, anti-dripping agent) and formulation procedures (pre-dispersion of solid additives in polyol via milling) which influence the flame retardancy of 6,6′-[ethan-1,2-diylbis(azandiyl)]bis(6H-dibenzo[c,e][1,2]oxaphosphin-6-oxid) (EDA-DOPO) containing flexible polyurethane foams has been investigated in this work. For comparison, the flame retardancy of two additional structurally-analogous bridged 9,10-dihydro-9-oxa-10-phosphaphenanthrene-10-oxide (DOPO)-based compounds, i.e., ethanolamine-DOPO (ETA-DOPO) and ethylene glycol-DOPO (EG-DOPO) were also evaluated together with EDA-DOPO in flexible PU foams of various formulations. The flame retardancy of these three bridged-DOPO compounds depends on the type of PU formulation. For certain PU formulations containing EDA-DOPO, lower fire performance was observed. Addition of emulsifier and polytetrafluoroethylene (PTFE) to these PU formulations influenced positively the flame retardancy of EDA-DOPO/PU foams. In addition, dispersion of EDA-DOPO and PTFE via milling in polyol improved the flame retardancy of the PU foams. Mechanistic studies performed using a microscale combustion calorimeter (MCC) and its coupling to FTIR showed no difference in the combustion efficiency of the bridged-DOPO compounds in PU foams. From MCC experiments it can be concluded that these bridged-DOPO compounds and their decomposition products may work primarily in the gas phase as flame inhibitors. The physiochemical behavior of additives in PU formulation responsible for the improvement in the flame retardancy of PU foams was further investigated by studying the dripping behavior of the PU foams in the UL 94 HB test. A high-speed camera was used to study the dripping behavior in the UL 94 HB test and results indicate a considerable reduction of the total number of melt drips and flaming drips for the flame retardant formulations. This reduction in melt drips and flaming drips during the UL 94 HB tests help PU foams achieve higher fire classification.

## 1. Introduction

Polyurethanes are inherently flammable materials and their flammability is generally reduced by addition of flame retardant additives [1]. The influence of addition of flame retardant additives in the thermal decomposition process as well as in improving their flame retardancy is well studied [1,2,3,4]. PU foams are manufactured using a variety of raw materials and process additives. However, little has been reported which reflects the influence of these materials on thermal decomposition and fire behavior of the polyurethanes. It is known that increased concentration of some PU components (silicone additives and metal catalysts) in the PU foams have a detrimental effect on fire behavior. It has also been found that MDI based PU foams display better fire performance compared to that of TDI based PU foams and the chemistry of polyols additionally influences the fire performance of the PU foams [5].

The need to replace toxic halogenated flame retardants has stimulated a rapid increase in the development of safer and effective alternative flame retardants. 9,10-Dihydro-9-oxa-10-phosphaphenanthrene-10-oxide (DOPO) derivatives have shown a great promise due to their versatile application in polymers [6]. Numerous derivatives of DOPO such as phosphonate [7,8], phosphinates [7,9] and phosphonamidates [6,10] have been utilized as flame retardant additives. DOPO derivatives were found to be very effective in flame retardation of thermoplastic and thermoset polymers. The P-H bond of DOPO allows for easy transformation into derivatives offering varying physical and chemical properties [11]. Bridged DOPO derivatives are very interesting due to their high thermal stability, effectiveness, ease of synthesis and possible commercial exploitation [6].

Flame retardant application of a DOPO based bridged phosphonamidate compound EDA-DOPO on flexible PU foams has been reported [6,10]. The bridged phosphonamidate compound was proven to be more effective compared to the analogous mono-phosphonamidate compounds [6,10,11]. EDA-DOPO has been recently REACH registered in Europe by FoamPartner AG, Switzerland and is currently industrially produced by Metadynea, Austria. Other analogous bis-DOPO compounds (ETA-DOPO and EG-DOPO) have been synthesized and their toxicity behavior investigated [12]. In a battery of in-vitro test systems (representing the human nervous system, the skin, and the lung) ETA-DOPO and EG-DOPO displayed adverse effects at concentrations > 10 µM. In contrast, EDA-DOPO was non-toxic to all cell types tested and, furthermore, did not adversely influence algae growth or daphnia viability [12]. Thus, it is clear that the structure of such DOPO compounds determines their toxicity behavior; however, it is not clear if small differences in their chemical structure may influence their fire behavior. Knowledge of fire performance behavior of these analogous bridged-DOPO compounds will facilitate the development of efficient flame retardant materials and their possible commercial exploitation.

The main objective of this work was to understand the influence of various additives and PU formulation procedure on fire performance of PU foams. Three bridged DOPO compounds, namely 6,6′-(ethane-1,2-diylbis(azanediyl))bis(6H-dibenzo[c,e][1,2]oxaphosphinine-6-oxide) (EDA-DOPO), 6-(2-((6-oxido-6H-dibenzo[c,e][1,2]oxaphosphinin-6-yl)amino)ethoxy)-6H-diben-zo[c,e][1,2]oxaphosphinine 6-oxide (ETA-DOPO), and 6,6′-(ethane-1,2-diylbis(oxy))bis(6H-dibenzo[c,e][1,2]oxaphosphinine-6-oxide) (EG-DOPO) were synthesized with good yields (>80%) and incorporated in different PU formulations. The foams were evaluated for fire performance behavior using the UL 94 HB test. The flame retardant efficacy of the bridged-DOPO compounds was studied using thermogravimetric analysis (TGA) and using microscale combustion calorimetry (MCC) coupled to an FTIR. Further modified formulations containing EDA-DOPO were developed and analyzed for their fire performance using the UL 94 HB test. These modified formulations involved incorporation of an anti-dripping agent and emulsifier. The effect of dispersion of EDA-DOPO and anti-dripping agent on their flame performance was also evaluated. The melt dripping behavior of the foams was also studied in the UL 94 HB tests using a high-speed camera.

## 2. Experimental

### 2.1. Materials and Methods

All chemicals for the synthesis of bridged-DOPO compounds were purchased from Sigma-Aldrich, Switzerland and were used without further purification. Chemicals used in the synthesis of PU foams: PO 56 (a propylene oxide polyol with a molecular weight of 3000 g/mol and a hydroxyl value of 56 KOH/g), Soft 38(a EO rich polyol with a molecular weight of 4500 g/mol and hydroxyl value of 38 mg KOH/gram), TDI 80 (a 80:20 mixture of 2,4-toluene diisocyanate and 2,6-toluene diisocyanate), urea, tin(II) octanoate catalyst, triethylenediamine, silicone surfactant, and sodium dodecyl sulfonate, were supplied by FoamPartner AG, Wolfhausen, Switzerland and were used without further purification. 9,10-dihydro-9-oxa-10-phosphaphenanthrene-10-oxide (purity 98%) was bought from Metadynea, Austria. PTFE with various particle sizes P1 (1 µm), P4 (4 µm), P8 (8 µm), and P40 (40 µm) were supplied by 3M, Dyneon GmbH, Burgkirchen an der Alz, Germany. 

The bulk density of synthesized additives was measured following the ASTM standard testing procedure (ASTM D7481-09). The parameters of the NMR spectroscopy (Bruker AV-III 400 spectrometer, Bruker Biospin AG, Fällanden, Switzerland) are summarized in the Supporting Information and fully assigned ^1^H, ^13^C, and ^31^P NMR spectra are shown in Figure S1a–f, (Supplementary Materials). 6,6′-(ethane-1,2-diylbis(azanediyl))bis(6H-dibenzo[c,e][1,2]oxaphosphinine-6-oxide) (EDA-DOPO), 6-(2-((6-oxido-6H-dibenzo[c,e][1,2]oxaphosphinin-6-yl)amino)ethoxy)-6H-dibenzo[c,e][1,2]oxaphosphinine 6-oxide (ETA-DOPO), and 6,6′-(ethane-1,2-diylbis(oxy))bis(6H-dibenzo[c,e][1,2]oxaphosphinine-6-oxide) (EG-DOPO) were synthesized according to the procedure as described in the literature[12]. The diastereomer ratio of EDA-DOPO has been reported as (1:1) [6,11] however, a more deep insight into the microstructure for EDA-DOPO in this study shows the diastereomer ratio to be (1.2:0.8).

The structure, physical and thermal properties of these bridged-DOPO compounds are summarized in Table 1. 

### 2.2. PU Foam Manufacturing

The PU foams were manufactured as per the base recipes (formulation A and B) described in Table 2, with modified B formulations described in Table 3. All foams were manufactured based on 300 grams of PO 56 polyol. In the case of blank foams (without FR additive), the required quantities of the polyol, water, catalysts, and surfactant were weighed accordingly in a plastic beaker (1100 mL volume). Components in the plastic cup were mixed with a mechanical stirrer (Heidolph Instruments GmbH & CO. KG, Schwabach, Germany) equipped with a Visco Jet type impeller [6] for 90 s at 950 rpm. Subsequently, the required quantity of TDI was added to the plastic beaker under continuous stirring (6 sec) of the reaction components. The mixture was then immediately transferred into the box and allowed to rise freely. Approximately 3 min after the termination of the foam growth process, the foam sample was placed in an oven (drying cabinet) for 1 h, at 80 °C. The cured foam was removed from the box and left to cool down at room temperature, conditioned for 48 h at 25 °C and 50% of relative humidity. After conditioning, the foams were cut into the required dimensions for further analysis.

For the preparation of the foams with solid additives (i.e. bridged DOPO derivatives and PTFE) two separate dispersion approaches were followed. According to the first procedure (formulation A), the solid additive (bridged DOPO compounds) was manually milled in a mortar and sieved on a set of vibrating sieves to the size ≤ 250 µm. Thereafter, the solid FR dispersion in polyol was obtained by mixing the required amount of the solid additive in a polyol/water mixture by using a high-speed digital homogenizer (Ultra Turrax T18 from IKA) for 5 min at a speed of ~9000 rpm. In the second procedure (formulations BDisp and BP described in Table 3), the dispersion of the solid additives in polyol was performed using a planetary ball mill (mono mill PULVERISETTE 6 from FRITSCH). In addition to the BP foams manufactured via milling methodology, PTFE containing foams using normal dispersion procedure as described for foams from formulation A and B were also manufactured. The solid additives were placed in a mill and dry ground for 30 min (three cycles, 10 min long each). The required amount of polyol was added to the mill and wet ground for 4 h (12 cycles, 20 min long each).

Subsequently, the required amounts of other ingredients of the PU foams were added and foaming was performed as described earlier for the blank foams. All manufactured PU foams had densities of 50 ± 3 kg/m^–3^.

Foams using BE the formulation (Table 3) were prepared according to the method described for formulation A foams (Table 2), however, no Soft 38 was added.

### 2.3. UL 94 HB Tests

The PU foams were evaluated for their flammability according to the UL 94 HB test where the specimen is tested in a horizontal orientation and a cotton web is placed underneath. In this test, the flame is applied to the free end of the specimen for 60 seconds and then removed. The 150 mm long test specimen is marked on the 25, 60 and 125 mm positions and the burning rate is measured between a 100 mm span. The sample size is 150 × 50 × 13 mm^3^ (length × width × thickness). The corresponding classification was made according to the procedure reported in the literature [13]. A minimum of five specimens were tested for each PU formulation. 

The dripping behavior of the PU foams in UL 94 HB test was documented by a high-speed Casio Exilim EX-F1 camera (CASIO Europe GmbH, Norderstedt, Germany). The camera was placed perpendicular to the foam sample length and the movies were recorded at 300 frames/sec. For these test series, the above-mentioned cotton web was not used since its burning interfered with the recording of the data. After the measurements, the videos were manually analyzed for the number of burning and non-burning drips via visual inspection. Five specimens of each material were tested for the drip test and average data is reported. 

### 2.4. Thermal Analysis

Thermogravimetric analysis (TGA) of the PU foam and bridged DOPO compounds was carried out by using a NETZSCH TG209 F1 Iris instrument (Waldkraiburg, Germany). The sample (exact weighing of approx. 3 mg) was heated from 25 to 800 °C at a heating rate of 10 °C·min^–1^. The measurements were performed two times under a nitrogen atmosphere with a total gas flow of 50 mL·min^–1^.

The melting point of the bridged-DOPO compounds was analyzed using a NETZSCH DSC Polyma 214 (Waldkraiburg, Germany). Samples (ca. 2–3 mg) in closed crucibles were heated to 300 °C at a heating rate of 10 °C·min^–1^. 

### 2.5. MCC/FTIR Coupling

Microscale combustion calorimetry (MCC) was used to assess the flammability of small samples (2–3 mg) of PU foam. Typically, the sample is heated under nitrogen flow at 1°C/s up to 750 °C. The released pyrolytic gases are extracted to the combustor where they are burnt. Pyrolysis and combustion are well separated and the parameters such as temperature and oxygen content could be controlled in the combustor allowing a partial oxidation of gases. The heat release is calculated via an oxygen analyzer according to Huggett’s relation (1 kg of oxygen consumed releases 13.1 MJ of heat). The heat release rate (HRR in W/g) as a function of the temperature and the total heat release (THR in kJ/g) was determined. MCC tests were performed using a Fire Testing Technology apparatus. A Nicolet iS10 FTIR spectrometer from ThermoFisher Scientific (Waldkraiburg, Germany) was coupled to the MCC exhaust (or to the combustor exhaust) via a heated transfer line. The temperatures of the transfer line and of the gas cell were fixed at 200 and 165 °C, respectively. FTIR spectra (range of 400–4000 cm^–1^, resolution fixed to 0.5 cm^–1^) were studied using Omnic software from ThermoFisher Scientific.

## 3. Results and Discussion

### 3.1. PU Foam Formulations

Standard PU formulations (A and B) as described in Table 2 were chosen in this work. Formulation B is a simplified version of formulation A in which Soft 38 and an emulsifier (sodium dodecyl sulfonate) are omitted. Unlike PO 56 which is mostly propylene oxide polyol, Soft 38 is an ethylene oxide rich polyol and used as a processing aid in the formulation. The emulsifier is normally added to improve mixing of the two polyols due to their different polarity. The simplified B formulation is industrially more relevant as it uses fewer amounts of process additives and offers long-term process stability in an industrial production. PU foams obtained using formulation A have been manufactured and well characterized [6,13,14], however, the flame retardant properties of PU foams composed of formulation B are yet unknown. This work emphasizes manufacturing and characterization of foams from formulation B.

Table 3 details modified B formulations containing EDA-DOPO as the flame retardant (FR) additive. In formulation BDisp, EDA-DOPO is dispersed by use of planetary ball milling prior to the foaming process. It is expected that such pretreatment of EDA-DOPO will improve its dispersion in polyol and increase its fire performance [6]. In formulation BE sodium dodecyl sulfonate, which is originally present in formulation A, was incorporated and expected to improve the EDA-DOPO dispersion. In formulation BP, PTFE of various sizes as an anti-dripping agent was incorporated.

### 3.2. Properties of Bridged DOPO Compounds

The three bridged DOPO compounds were synthesized in good yields (81%, 82%, and 92% for EG-DOPO, ETA-DOPO, and EDA-DOPO, respectively) according to the procedure as described in the literature [12]. The structure and the properties of the bridged DOPO compounds are summarized in Table 1. The obtained compounds are white powders with similar bulk density. However, they exhibit varying thermal properties. EDA-DOPO exhibits the highest melting point and *T*_d_ 5% which may be due to strong amide interactions between the O=P–N– groups. The melting points of the compounds estimated via DSC measurements show wide melting peaks which could be due to the difference in the melting behaviors of the diastereomers. Owing to the chirality of the phosphorus stereocenter of DOPO, two sets of diastereomers can be obtained in its corresponding bridged-derivatives and can be differentiated by NMR spectroscopy. The NMR analysis of EG-DOPO and ETA-DOPO showed the existence of two set of diasteromers (SR + RS and SS + RR) in equimolar ratios (50: 50% each) (Figure S1a–d, respectively) and for EDA-DOPO a ratio of about 60: 40% was evaluated (Figure S1e,f, Supplementary Materials). The thermal decomposition data (*T*_d_ 5%) of the three bridged DOPO compounds (Table 1 and Figure S2, Supplementary Materials) indicate EDA-DOPO (357 °C) to be the most stable, followed by EG-DOPO (341 °C) and ETA-DOPO (321 °C). The TGA data showed that EDA-DOPO forms 7% of char residue (Figure S2, Supplementary Materials) at 800 °C while both of the other bridged DOPO compounds form no residue at the same temperature. 

### 3.3. Effect of PU Formulation on Fire Performance

Table 4 summarizes the UL 94 HB results for foams obtained from formulations A and B. Blank foams of both formulations did not show any UL 94 HB classification since they were entirely burned and produced flammable drips without leaving any residue. It is clear from the results that the foams of formulation A containing bridged DOPO compounds have the highest UL 94 HB rating of HF1 even at low concentrations of 2.5% (amount of DOPO FR used based on the weight of polyol). All bridged DOPO compounds produced from formulation A showed the same level of fire performance. However, the fire results of foams from formulation B clearly demonstrate the difference in the fire performance among the three bridged compounds. 

In general, the fire performance of all bridged compounds at 2.5% concentration in formulation B foams was lower compared to analogous foams from formulation A. Unlike in the case of formulation A, a minimum concentration of 5% for EG-DOPO and ETA-DOPO is needed to achieve the highest UL 94 HB classification of HF1. Foams containing EDA-DOPO could only achieve HF2 rating even at 7.5% concentration. Normally five specimens are tested for each formulation and all five specimens should have identical fire classification. If for any reason all five specimens do not achieve the same classification, one can repeat the fire test with 5 new specimens. The final UL 94 HB classification is always the lower rating among the five specimens tested in a set. Foams made from formulations B containing 5% EDA-DOPO were tested four times (overall, four sets of five specimens) and only 30% specimens could achieve the HF1 ratings. Similarly, 7.5% EDA-DOPO containing foams from formulation B was tested 5 times (overall 5 sets of 5 specimens) and a higher percentage% (55%) of specimens could achieve HF1 rating.

Increasing the concentration of EDA-DOPO, therefore, improves the fire performance of the foams of formulation B; however, there is a variation in the fire performance and only a lower overall rating of HF2 can be achieved. It can be inferred that ETA-DOPO and EG-DOPO seem to have higher flame retardant efficacy in formulation B compared to EDA-DOPO.

To further understand the possible reasons behind this difference in the fire performance of the three bridged DOPO additives, thermal and combustion analysis of the PU foams was performed. The TGA data of the PU foams (Figure S3, Supplementary Materials) from formulation B clearly shows that the addition of bridged DOPO compounds has a negligible effect in the condensed phase. The thermal decomposition profile of foams containing the FRs is similar to the blank foam. There is a slight improvement in the thermal stability of the PU foam in the first stage of decomposition (200–300 °C) and is similar to the published data [6]. Addition of the bridged DOPO compounds only slightly improves the char residue for the foams and the cone calorimetry data showed that EDA-DOPO primarily acts in the gas-phase [6] and it is expected that the two analogous bridged DOPO compounds (ETA-DOPO and EG-DOPO) behave similarly in fire.

It is, thus, hypothesized here that there may be a difference in the gas phase activity of the three bridged DOPO additives in formulation B. It has been shown that the use of microscale combustion calorimetry (in an incomplete combustion scenario) is an interesting tool for assessing the gas phase activity of flame retardants [15,16,17,18]. In order to investigate the gas phase activity of the bridged DOPO compounds, the combustion efficiency of foams was studied using MCC and MCC/FTIR coupling. It can be shown that the decrease of the combustor temperature Tc in MCC leads to an incomplete combustion [17,18]. The corresponding decrease of combustion efficiency χ(*T*_c_) can be used as an indicator of flame retardant gas phase activity. It was proposed that the combustion efficiency curve could be described by an Arrhenius law [15]. This permits determining the activation energy of the oxidation reaction that can be used to quantify the gas phase activity of FRs. Following the procedure described in the literature [18], MCC tests were performed at 600, 650, 700, and 900 °C on PU foams containing the DOPO derivatives at a 15% weight loading. A rather high concentration of the bridged DOPO compounds (15%) in the PU foam to magnify the possible gas phase flame inhibition effect was used in this work. It should be mentioned that all HRR curves (not presented here) exhibit two peaks related to the two-step decomposition of the PU foam. The shape of the curves was altered neither by the DOPO compounds nor by the combustion temperature. The main effect of the combustion temperature variation was to decrease the overall HRR signal. Considering that the combustion was complete at 900 °C, the combustion efficiency at a given combustion temperature χ(*T*_c_) was calculated by dividing the THR at *T*_c_ by the THR at 900 °C. 

**Figure 1:**
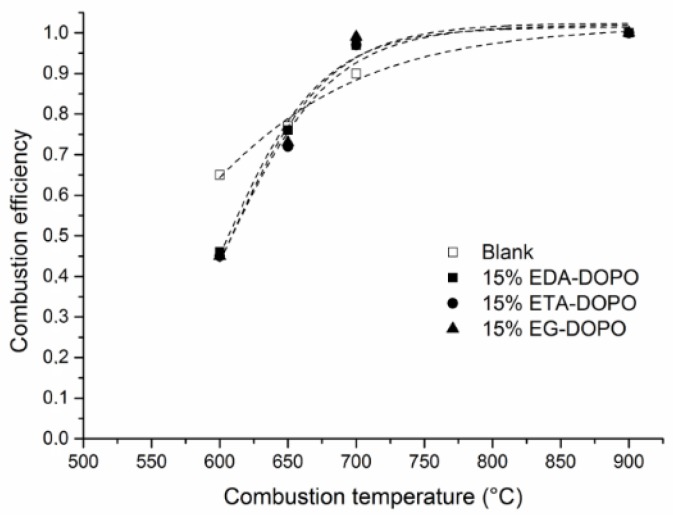
Combustion efficiency versus of combustor temperature for the three flame retarded foams.

Figure 1 shows the change in combustion efficiency as a function of combustion temperature. It is noted that the combustion efficiency starts decreasing at combustion temperature below 700 °C. For the blank foam, the decrease of χ (*T*_c_) is relatively moderate and reaches 65% at 600 °C whereas the combustion efficiency for the flame retarded foams decreases more markedly down to 45% at 600 °C. This result reflects the gas phase activity of DOPO derivatives. Nevertheless, no significant difference between the three flame retardants could be highlighted.

Further analysis of combustion gases was performed using a MCC/FTIR coupling. It was shown in a previous paper [19] that, by varying the combustor temperature of the MCC, this coupling enables characterizing and quantifying the gases of incomplete combustion such as carbon monoxide, methane, ethylene or acetylene. It was also reported that the analysis of combustion gases exiting from MCC could help understanding the gas phase combustion inhibition of some flame retardants [15]. 

**Figure 2:**
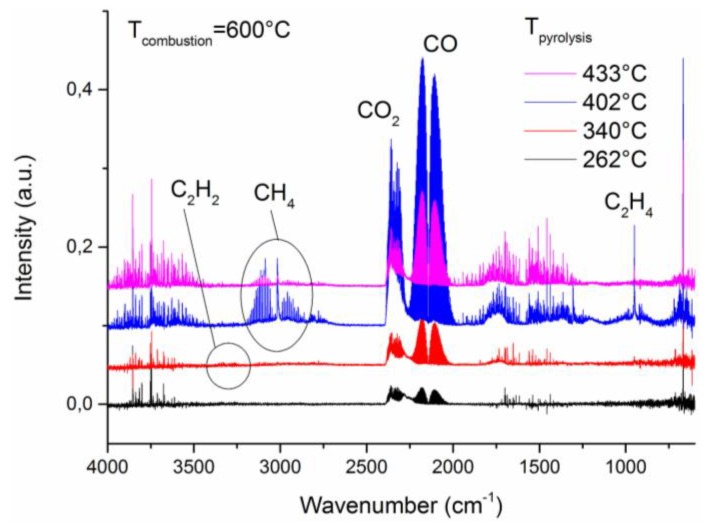
FTIR spectra of gases exhausted from MCC after pyrolysis of 15% EDA-DOPO at various pyrolysis temperatures and under combustion conditions at 600 °C.

Figure 2 shows FTIR spectra of combustion gases collected at different pyrolysis temperatures and a given combustion temperature of 600 °C for 15% EDA-DOPO. The presence of CO bands (2250–2000 cm^–1^) confirms incomplete combustion at 600 °C. Furthermore, the presence of additional gases can be detected. Bands of methane (regions 3240–2810 and 1400–1250 cm^–1^), as well as specific bands of ethylene (1150–800 cm^–1^), were identified. Traces of acetylene might be responsible for the bands at 3400–3200 cm^–1^. No phosphorus compounds have been detected by means of this FTIR analysis. This could be ascribed to possible condensation or adsorption of phosphorus species in the transfer line which is kept a relatively lower working temperature of 200 °C. An attempt was made to perform measurements with the transfer line temperature of 300 °C, but it was not successful.

**Figure 3:**
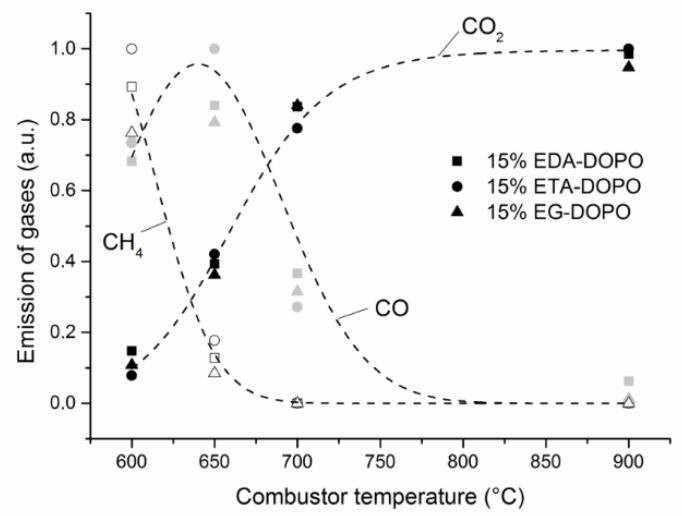
Emission of CO_2_, CO, and CH_4_ versus combustor temperature.

By integrating FTIR bands specific to CO_2_, CO, and methane, it was possible to follow the relative emission of these gases according to the combustor temperature (Figure 3). At 900 °C complete combustion is observed and CO_2_ is the only carbon-containing combustion product. At lower temperatures, the combustion becomes incomplete and CO emission increases at the expense of CO_2_ showing a maximum at circa 650 °C. This is due to the fact that at temperatures below 650 °C the combustion is incomplete and non-oxidized species such methane can be found in the exhausted products. It is noteworthy that the three flame retardant foams behave almost similarly and, therefore, it is concluded that the gas phase action of the three DOPO derivatives must be nearly identical. 

It is thus very likely that the difference in the flame retardant behavior of the PU foams from formulation B must be somehow related to the composition of the PU foam. Possible reasons for this observation could be a difference in the dispersion of the FR additives in the polyol or physical and chemical interaction of several PU components during the PU thermal decomposition process. 

It has been confirmed via in vitro tests using standard assays that EDA-DOPO is relatively non-toxic compared to the other two bridged DOPO compounds which were found to be very toxic in some in vitro tests [12]. For any future commercial exploitation of a new FR additive, it needs to not only exhibit relatively low toxicity but it should also have high flame retardant efficacy. Thus following efforts were made to further optimize formulation B containing EDA-DOPO so as to achieve higher level of flame retardancy, similar to the level shown for foams from formulation A. Table 3 shows the various approaches undertaken in this work so as to improve the fire performance of formulation B. Three main strategies were followed: (1) improved dispersion of EDA-DOPO via use of an emulsifier, (2) improved dispersion of EDA-DOPO via ball milling in polyol, and (3) use of PTFE as an anti-dripping agent. The rationale behind the first two approaches was that an improved dispersion of solid additives in polyol would have a beneficial effect on the reproducibility of fire results. Use of PTFE as an anti-dripping agent is a common practice in thermoplastics, however, relatively less is known about their application in PU foams. Flexible PU foams exhibit dripping behavior when exposed to flame [13,14,20], thus, strategies in reducing the number of flaming drips may increase the chance of obtaining a HF1 classification in UL 94 HB tests.

### 3.4. Modification of Formulation B and Fire Performance

Table 5 summarizes the UL 94 HB fire results of foams obtained via formulation B. Industrial PU manufacturing processes normally involves mixing streams of polyols and isocyanates to obtain slab stock PU foam. Additives are normally added to the polyol component before reaction with the isocyanates. Normally liquid additives are preferred ingredients in the PU manufacturing process due to the ease of handling and good mixing. In the case when solid additives are used, they need to be dispersed well for uniform functional properties [21]. It has been demonstrated that a proper dispersion of additives in the polyol is necessary for long-term storage stability of bridged DOPO polyol dispersions [22]. Thus in an attempt to improve the flame retardancy of EDA-DOPO in formulations B, pre-dispersion of EDA-DOPO in polyol using a planetary ball mill was performed. In the UL 94 HB fire testing, the major difference in the classification HF2 and classification HF1 is the allowance of the burning of a cotton web. In HF1 rating the cotton underneath the PU foam shouldn’t burn. In the case when EDA-DOPO is incorporated in the polyol using a high-speed homogenizer, the UL 94 HB results for formulation B is HF2 (Table 4). In this case, all five specimens had HF2 ratings. However, in case when ball milling is used for dispersion of EDA-DOPO (Table 3) in formulation B there is an improvement of flame retardancy of the PU foams. As seen in Table 5 the 30% and 60% of the foam specimens could pass the HF1 ratings for 2.5% and 5% EDA-DOPO concentrations, respectively. A clear HF1 rating for foams containing 6% EDA-DOPO concentration in formulation B was achieved. HF 1 rating was not possible even at 7.5% EDA-DOPO concentration when using only a high-speed homogenizer for its dispersion (Table 4).

**Figure 4:**
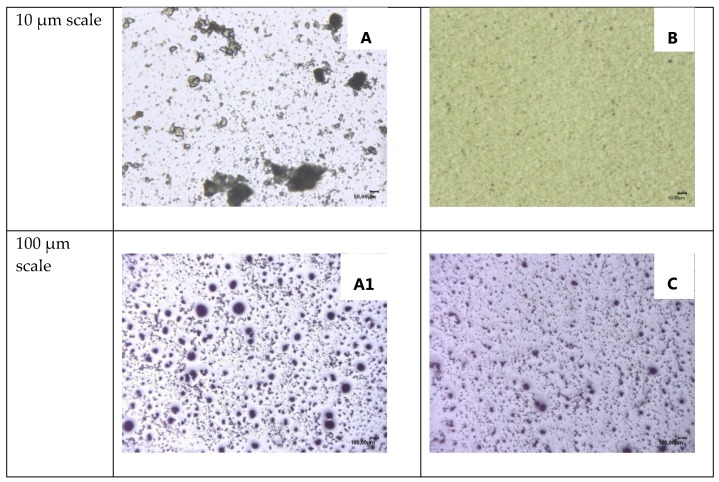
Optical images of EDA-DOPO dispersions in the polyol: (**A**, **A1**) 5% EDA-DOPO dispersed in polyol using high speed homogenizer, (**B**) 5% EDA-DOPO dispersed in the polyol using a planetary ball mill, (**C**) 5% EDA-DOPO dispersed using sodium dodecyl sulfonate in polyol using high-speed homogenizer.

The dispersion of EDA-DOPO in polyol was further analyzed using an optical microscope (Figure 4). Picture A shows the presence of very large (>10 µm) particles or agglomerates of EDA-DOPO whereas picture B shows a fine (1–2 µm) and uniform distribution of EDA-DOPO in the polyol matrix. It is believed that the use of milling for dispersion of EDA-DOPO ensures its uniform distribution in cell walls of PU foams and thus improves the fire performance of the PU foams.

One of the key differences in formulation A and B was the use of sodium dodecyl sulfonate as an emulsifier. Thus, after it was incorporated in the formulation B, a clear improvement in the fire performance as seen in Table 5 was observed. A clear HF1 rating could be achieved with 5% EDA-DOPO, whilst 60% of the specimen group of 2.5% EDA-DOPO concentration in formulation B foam attained an HF1 Rating. It seems that the addition of sodium dodecyl sulfonate also improves the dispersion of EDA-DOPO in the polyol which is confirmed by the entry C in Figure 4. Adding sodium dodecyl sulfonate reduces the number of larger agglomerates (>50 µm) of EDA-DOPO (entry A1, Figure 4). Additionally, the inclusion of sodium dodecyl sulfonate has higher improvement in the fire performance (a clear HF 1 at 5% EDA-DOPO concentration) compared to ball mill dispersion method (HF2 at 5% EDA-DOPO concentration) even though the latter has a better dispersion and reduction in particle size (<5 µm) of EDA-DOPO in polyol. Thus, one can conclude that better dispersion of EDA-DOPO does have a beneficial effect on the fire performance of PU foam. However, the addition of sodium dodecyl sulfonate may have an auxiliary effect on flame retardancy of PU foams which is not clear at this stage.

PTFE is commonly used as anti-dripping agent [23,24] and as synergist [25,26,27] in flame retardant formulations of polymers. Unlike halogenated flame retardants, PTFE works in the condensed phase by reducing the melt flow of the polymer and dripping behavior [28]. It is also known to reduce flaming drips [29] and is normally added to engineering plastics to improve their fire performance in UL 94 tests, however, its use in PU foam flame retardancy is relatively unknown. It was observed for the foams obtained from formulation B containing EDA-DOPO that the fire results were inconsistent (HF2 vs HF1), and consequently it was hypothesized that a small addition of PTFE to the formulation B could improve its flame retardancy. As shown in Table 5, an addition of 0.5% PTFE to the formulation helped achieve a HF1 rating for 7.5% EDA-DOPO formulation. This formulation was obtained by normal dispersion with a high-speed homogenizer. Lower amounts of PTFE (below 0.5%) did not have any influence in improving the flame retardancy while using this procedure. At PTFE concentrations lower than 0.5%, the same level of flame retardancy was obtained as that for reference foams (EDA-DOPO foams from formulation B). 

The effect of particle size of PTFE and milling pretreatment for dispersion on flame retardancy of the PU foams was further studied. As seen in Table 5, the dispersion of solid additives via milling in polyol remarkably influences the flame retardancy of the respective foams. In this case, 0.5 % PTFE and 5% EDA-DOPO is needed to achieve HF1 classification which was not possible using homogenizer mixing (HF2 rating for 5% EDA-DOPO). Thus, dispersion of PTFE and EDA-DOPO using milling is beneficial in achieving a higher UL 94 classification with a lower EDA-DOPO concentration. The addition of PTFE alone in the formulation B foams had no improvement in fire performance of the PU foams. It is also observed from Table 5 that the particle size of the PTFE manifests some influence on the fire performance of the PU foams. PTFE with larger particles (40 µm) offers no improvement in the fire performance of the PU foam. The smaller particle size of additives offers higher specific surface area and, thus, ensures better uniform functional properties. 

### 3.5. Dripping Behavior of PU Foams during UL 94 HB Tests

As described earlier in previous sections it was observed that some PU formulations gave fire results which were not consistent and it hypothesized that a clear understanding of the dripping behavior in UL 94 HB testing would provide more insights in the burning behavior of the foams and help explain the difference in flame retardant efficacy of the additives. A high-speed camera was used to monitor the number of flaming and non-flaming drips, with the results of these experiments summarized in Table 6. During these experiments, cotton web was not placed underneath the PU foams to avoid any complications with video analysis later. If the cotton web was placed underneath the PU foam, the ignition of the cotton web made the video analysis difficult.

As expected, the blank foams of formulation A and B had the highest number of drips and flaming drips. Addition of flame retardants to the formulation A significantly reduced the number of drips and flaming drips. 5% EDA-DOPO A foams exhibited approximately 50% lower amount of drips and almost no burning drips, thus achieving the highest fire classification. However, the number drips and flaming drips remained similar in case of 5% EDA-DOPO B compared to the blank B foams thus achieving a lower reacting of HF2. As discussed earlier, 5% ETA-DOPO B which achieves HF1 classification exhibits significantly lower drips and non-flaming drips in the fire test. One can speculate the lower melting temperature of ETA-DOPO (~178 °C) could help it distribute better in the molten drips compared to EDA-DOPO (~275 °C) which has a significantly higher melting point. The dripping results of EG-DOPO foams from formulation B were similar to that of 5% ETA-DOPO B foams and thus not reported in Table 6.

Addition of sodium dodecyl sulfonate in 5% EDA-DOPO BE foam considerably reduces the number of drips and non-flaming drips. Further dispersing EDA-DOPO via milling in the polyol (i.e., 5% EDA-DOPO BDisp) has similar dripping behavior. An higher rating of HF1 for 5% EDA-DOPO BE foams clearly indicates an additional benefit of using sodium dodecyl sulfonate which is not very clear. However, dispersion of EDA-DOPO is important in obtaining consistent fire results with sodium dodecyl sulfonate playing a more effective role in improving the flame retardancy of EDA-DOPO foams. Addition of sodium dodecyl sulfonate to the blank B foams did not have any significant influence on the fire performance of the foam.

## 4. Conclusions

In this work some key factors which affect the flammability of flexible PU foams was identified and studied. Three different structurally analogous bridged DOPO compounds namely EDA-DOPO, ETA-DOPO, and EG-DOPO were investigated for their flame retardant behavior in foams obtained from two different PU formulations. There was no difference in the flame retardant effect (UL 94 HB tests) of the three additives in formulation A; however, EDA-DOPO had a lower fire performance compared to the other two bridged compounds for foams of formulation B. In an earlier work EDA-DOPO has been shown to be non-toxic compared to the other two bridged DOPO compounds, REACH registered in Europe and, thus, a choice of current commercialization. Thus, further work was carried out to understand this difference in flame retardant efficacy of the three DOPO additives and improve the fire performance of EDA-DOPO in formulation B. The thermal analysis of the PU foams and subsequently combustion analysis via MCC and MCC/FTIR coupling indicated no difference in the gas phase flame inhibition effect of the three bridged DOPO additives. Further modification of formulation B containing EDA-DOPO was performed via three approaches, namely; (1) improved dispersion of EDA-DOPO via the use of an emulsifier, (2) improved dispersion of EDA-DOPO via ball milling in the polyol, and (3) use of PTFE as an anti-dripping agent. Use of sodium dodecyl sulfonate as an emulsifier and milling of EDA-DOPO in polyol significantly improved the dispersion of EDA-DOPO in the polyol and subsequently the fire performance of resulting foams in the UL 94 HB test. The improvement of EDA-DOPO dispersion in polyol by use of sodium dodecyl sulfonate as an emulsifier tend to give better fire results compared to ball milling of EDA-DOPO. The exact role of such an emulsifier in improving the fire performance behavior is not clearly understood. As expected, the addition of PTFE significantly improved the fire performance of EDA-DOPO B formulation foams. The molten drip analysis of PU foams during the UL 94 HB test clearly demonstrates the beneficial effect of modified B formulations in improving their fire performance. All modification strategies for formulation B can help reduce the number of molten drips and flaming drips in the UL 94 HB fire tests for foams. Thus, it is demonstrated in this research that, by careful design of new additives and proper PU formulation, one can achieve the desired fire performance. In the future, the effect of emulsifier in fire performance enhancement will be further investigated and findings of this research will be extended to other PU formulations.

## Figures and Tables

**Table 1:** Structure, physical properties, and chemical characteristics of the bridged-DOPO flame retardants.

Properties	EDA-DOPO	ETA-DOPO	EG-DOPO
Structures	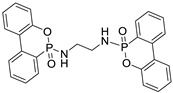	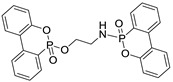	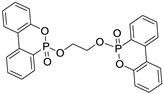
Appearance	White powder	White powder	White powder
Bulk density	2.10 g/mL	2.01 g/mL	2.02 g/mL
Melting point	272–275 °C	177–178 °C	130–147 °C
*T*_d_ 5% *	357 °C	321 °C	341 °C
%P	12.68	12.66	12.63
Diastereoisomers ratio	1.2:0.8	1:1	1:1

* *T*_d_ 5%: Temperature at 5% weight loss.

**Table 2:** PU foam formulations A and B.

Ingredients	Formulation A B	
	Part (grams)	
PO 56 (Polyol)	97	100
Soft 38 (Polyol)	3	-
Sodium dodecyl sulfonate	0.8	-
Stannous catalyst	0.25	0.45
Triethylenediamine	0.1	0.4
Silicone surfactant	0.5	0.5
Water (total)	1.85	1.6
TDI	29.8	26.2
FR ^a^	X ^b^	X ^b^

^a^ EDA-DOPO, ETA-DOPO, EG-DOPO; ^b^ Concentration of FR: 0%, 2.5%, 5%, 7.5%, 15% based on the weight of the polyol (PO 56).

**Table 3:** Modifications of formulation B.

Ingredients	Modified B Formulations		
	BDisp ^a^	BE	BP ^b^
	Part (grams)		
PO 56	100	100	100
Sodium dodecyl sulfonate	-	0.8	
PTFE	-	-	0.5 ^c^
Stannous catalyst	0.45	0.45	0.45
Triethylenediamine	0.4	0.4	0.4
Silicone surfactant	0.5	0.5	0.5
Water (total)	1.6	1.15	1.6
TDI	26.2	26.2	26,2
EDA-DOPO	0–7.5 ^d^	0–7.5 ^d^	0–7.5 ^d^

^a^ FR dispersion in Polyol (PO 56) prepared with a planetary ball mill; ^b^ PTFE: P1 (particle size 1 µm), P4 (particle size 4 µm), P8 (particle size 8 µm), P40 (particle size 40 µm); ^c^ Concentration of PTFE: 0.5% based on the total weight of the ingredients (excluding FR); ^d^ Concentration of FR: 0%, 2.5%, 5%, 7.5% based on the weight of the polyol (PO 56).

**Table 4:** UL 94 HB results for foams from formulations A and B.

Foam Samples	Formulation A	Formulation B
Conc. and FR Type	UL-94 HB	UL-94 HB
Blank	No Rating	No Rating
2.5% EDA-DOPO	HF-1	HF-2
5% EDA-DOPO	HF-1	**HF-2**/HF1 * (14/6)
7.5% EDA-DOPO	HF-1	**HF-2**/HF1 ** (11/14)
2.5% ETA-DOPO	HF-1	HF-2
5% ETA-DOPO	-	HF-1
7.5% ETA-DOPO	-	HF-1
2.5% EG-DOPO	HF-1	HF-2
5% EG-DOPO	-	HF-1
7.5% EG-DOPO	-	HF-1

* Twenty specimens were tested in a set of five each, 14 specimens achieved HF2 ratings and six specimens achieved HF1. Overall rating is HF2. ** Twenty-five specimens were tested in a set of five each, 11 specimens achieved HF2 ratings, and 14 specimens achieved HF1. Overall rating is HF2.

**Table 5:** UL 94 HB results for PU foams from Modified B formulations.

Type of Modified Formulations		FR Concentration	UL-94 HB
Dispersion via ball mill (**BDisp**)		2.5% EDA-DOPO	**HF-2**/HF1 * (7/3)
		5% EDA-DOPO	**HF-2**/HF1 ** (4/6)
		6% EDA-DOPO	HF-1
		7.5% EDA-DOPO	HF-1
Use of surfactant sodium alkane Sulfonate (**BE**)		2.5% EDA-DOPO	**HF-2**/HF1 ***(4/6)
		5% EDA-DOPO	HF-1
		7.5% EDA-DOPO	HF-1
0.5% PTFE (1 µm size) Normal dispersion ^1^		2.5% EDA-DOPO	HF-2
		5% EDA-DOPO	HF-2
		7.5% EDA-DOPO	HF-1
Use of PTFE and Ball mill dispersion (**BP**)	1 µm, 0.5%	5% EDA-DOPO	HF-1
	4 µm, 0.5%	5% EDA-DOPO	HF-1
	8 µm, 0.5%	5% EDA-DOPO	HF-1
	40 µm, 0.5%	5% EDA-DOPO	HF-2

* Ten specimens were tested in a set of five each, seven specimens achieved HF2 ratings and three specimens achieved HF1. Overall rating is HF2; ** Ten specimens were tested in a set of five each, four specimens achieved HF2 ratings and six specimens achieved HF1. Overall rating is HF2; *** Ten specimens were tested in a set of five each, four specimens achieved HF2 ratings and six specimens achieved HF1. Overall rating is HF2; ^1^ The foams were manufactured using normal dispersion procedure as described for base formulation A and B. The concentration of all additives are based on the weight of polyol taken for foaming.

**Table 6:** Dripping behavior analysis of PU foams.

Foams	Total Drops	Burning Drops	UL 94 HB Rating
Blank A	132 ± 36	96 ± 37	NA
Blank B	106 ± 16	35 ± 35	NA
5% EDA-DOPO A	56 ± 5	3 ± 2	HF1
5% EDA-DOPO B	101 ± 6	32 ± 7	HF2
5% ETA-DOPO B	61 ± 6	1 ± 0.3	HF1
5% EDA-DOPO BE	68 ± 13	7 ± 5	HF1
5% EDA-DOPO BDisp	60 ± 8	9 ± 5	HF2/HF1(4/6) *
5% EDA-DOPO BDisp 0.5% P1	38 ± 2	14 ± 7	HF1

* 10 specimens were tested in a set of five each, four specimens achieved HF2 ratings and six specimens achieved HF1. Overall rating is HF2; **A**: formulation A, **B**: formulation B, **BE**: formulation B containing sodium dodecyl sulfonate, **BDisp**: formulation B where the EDA-DOPO is dispersed by milling, **BDisp 0.5% P1**: formulation B where the EDA-DOPO is dispersed by milling and contains 0.5% PTFE of 1 µm size.

## Supplementary Materials

The following are available online at http://www.mdpi.com/2073-4360/10/10/1115/s1, Figure S1a. ^1^H, ^13^C, and ^31^P NMR spectra of EG-DOPO (DMSO-d_6_); Figure S1b. Regions of interest of ^1^H-^13^C HSQC (A, B), ^1^H-^13^C HMBC (C), and ^1^H-^1^H DQF-COSY (D) NMR spectra of EG-DOPO (DMSO-d_6_); Figure S1c. ^1^H, ^13^C, and ^31^P NMR spectra of ETA-DOPO (DMSO-d_6_); Figure S1d. Regions of interest of ^1^H-^13^C HSQC (A, B), ^1^H-^13^C HMBC (C), and ^1^H-^1^H DQF-COSY (D) NMR spectra of ETA-DOPO (DMSO-d_6_), Figure S1e. ^1^H, ^13^C, and ^31^P NMR spectra of EDA-DOPO (DMSO-d_6_); Figure S1f. Regions of interest of ^1^H-^13^C HSQC (A, B), ^1^H-^13^C HMBC (C), and ^1^H-^1^H DQF-COSY (D) NMR spectra of EDA-DOPO (DMSO-d_6_); Figure S2. TGA data of bridged DOPO compounds (N_2_ atmosphere); Figure S3. TGA data of PU foams containing 5% bridged DOPO compounds (N_2_ atmosphere).
