# Supplementary material for: Some Key Factors Influencing the Flame Retardancy of EDA-DOPO Containing Flexible Polyurethane Foams
Source: Polymers (Basel). 2018 Oct 9;10(10):1115. doi: 10.3390/polym10101115 (PMC6403544; doi:10.3390/polym10101115)

Some Key Factors Influencing the Flame Retardancy of EDA-DOPO Containing Flexible Polyurethane Foams

Agnieszka Przystas[1], Milijana Jovic[1], Khalifah A. Salmeia[1], Daniel Rentsch[2], Laurent Ferry[3], Henri Mispreuve[4], Heribert Perler[4], Sabyasachi Gaan[1,*]

[1]Additives and Chemistry, Advanced Fibers, Empa Swiss Federal Laboratories for Materials Science and Technology, Lerchenfeldstrasse 5, 9014 St. Gallen, Switzerland

[2]Laboratory for Functional Polymers, Empa Swiss Federal Laboratories for Materials Science and Technology, Überlandstrasse 129, 8600 Dübendorf, Switzerland

[3]Ecole des Mines d'Alès, Centre des Matériaux des Mines d'Alès (C2MA), 6 Avenue de Clavières, F-30319 Alès Cedex, France

[4]Foampartner, Fritz Nauer AG, Switzerland

*Correspondence: sabyasachi.gaan@empa.ch; +41587657611

All ^{1}H, ^{13}C and ^{31}P NMR spectra were recorded on a Bruker Avance III 400 NMR spectrometer (Bruker Biospin AG, Fällanden, Switzerland) at 400.2, 100.6, and 162.0 MHz, respectively. The 1D NMR spectra, as well as the ^{1}H-^{13}C HSQC, ^{1}H-^{13}C HMBC, ^{1}H-^{13}C HSQC-TOCSY, ^{1}H-^{1}H DQF-COSY, and ^{1}H-^{31}P HMBC 2D correlation NMR experiments used for the complete assignment of resonances were performed at 298 K using the Bruker standard pulse programs and parameter sets on a 5 mm CryoProbe™ Prodigy probe equipped with z-gradient applying 90° pulse lengths of 11.4 µs (^{1}H), 10.0 µs (^{13}C) and 12.0 µs (^{31}P). ^{1}H and ^{13}C chemical shifts (δ) in ppm are calibrated to residual solvent peaks (DMSO-d$_6$: δ = 2.49 and 39.5 ppm), the ^{31}P chemical shifts were referenced to an external sample with neat H_3PO_4 at 0.0 ppm. Since all reported compounds consist of two inseparable diastereomers the coupling patterns of the ^{1}H NMR spectra remain complex and no reliable J values could be extracted. Wherever possible, the ^{1}H, ^{31}P coupling constants are reported in Hz. For ^{13}C NMR data multiplicities *s* = quaternary carbon, *d* = CH, *t* = CH_2, and *q* = CH_3 are shown and ^{31}P, ^{13}C coupling constants are reported in Hz. Weak correlations observed in the 2D NMR experiments are assigned as "*w*". For EG-DOPO and ETA-DOPO nearly 1:1 mixture of diastereomers were found disabling the discrimination of the generally doubled set of ^{13}C signals of the individual species by the heights of carbon resonances as it was possible for EDA-DOPO.

EG-DOPO (2 isomers, ca. 1:1)

^{1}H NMR (400.2 MHz, DMSO-d$_6$) δ (ppm): 8.19 (m, J(H,P) = 6.2, 2H, H-5); 8.14 (m, 2H, H-8); 7.80 (m, 2H, H-4); 7.71 (m, J(H,P) = 14.4, 2H, H-2); 7.53 (m, J(H,P) = 3.6, 2H, H-3); 7.41 (m, 2H, H-10); 7.30 (m, 2H, H-9); 7.18 (m, 2H, H-11); 4.18 (m, 4H, H-13).

^{13}C NMR (100.6 MHz, DMSO-d$_6$) δ (ppm): 148.5 (sd, J(C,P) = 7.8, C-12); 136.2 (sd, J(C,P) = 7, C-6); 134.0 (dd, J(C,P) = 2.5, C-4); 130.8 (d, C-10); 129.7 (dd, J(C,P) = 9.4, C-2); 128.6 (dd, J(C,P) = 15.2, C-3); 125.8 (d, C-8); 125.0 (d, C-9); 124.6 (dd, J(C,P) = 11.8, C-5); 121.8 (sd, J(C,P) = 11.8, C-7); 121.3 (sd, J(C,P) = 179.4, C-1); 119.8 (dd, J(C,P) = 6.6, C-11); 65.2 (td, J(C,P) = 5.9/1.9, C-13).

^{31}P NMR (162.0 MHz, DMSO-d$_6$) δ (ppm): 9.9

^{1}H-^{13}C HMBC: H-2 → C-(1w, 4, 6); H-3 → C-(1, 4w, 5); H-4 → C-(2, 5w, 6); H-5 → C-(1, 3, 7); H-8 → C-(6, 10, 12); H-9 → C-(7, 11); H-10 → C-(8, 11w, 12); H-11 → C-(7, 9, 12); H-13 → C-(13); H-2 → C-(2, 4, 5); H-3 → C-(1, 3); H-4 → C-(2); H-5 → C-(1, 2, 6, 9); H-6 → C-(5, 7, 8, 9); H-8 → C-(6); H-9 → C-(5w, 6).

^{1}H-^{1}H DQF-COSY: H-2 → H-(3); H-3 → H-(2, 4); H-4 → H-(3, 5); H-5 → H-(4); H-8 → H-(9); H-9 → H-(8, 10); H-10 → H-(9, 11); H-11 → H-(10); H-13 → H-(14).

^{1}H-^{31}P HMBC: H-(2, 3, 5, 13) → P

EDA-DOPO (2 isomers, ca. 1.2:0.8)

Major isomer (60%)

^{1}H NMR (400.2 MHz, DMSO-d$_6$) δ (ppm): 8.14 (m, J(H,P) = 3.1, 2H, H-5); 8.10 (m, 2H, H-8); 7.77 (m, J(H,P) = 22.1, 2H, H-2); 7.70 (m, 2H, H-4); 7.50 (m, J(H,P) = 3, 2H, H-3); 7.39 (m, 2H, H-10); 7.27 (m, 2H, H-9); 7.15 (m, 2H, H-11); 5.75 (m, J(H,P) = 11.8, 2H, NH); 2.85 (m, 4H, H-13).

^{13}C NMR (100.6 MHz, DMSO-d$_6$) δ (ppm): 149.4 (sd, J(C,P) = 7.2, C-12); 135.9 (sd, J(C,P) = 6.7, C-6); 132.7 (d, C-4); 130.4 (d, C-10); 129.4 (dd, J(C,P) = 9.6, C-2); 128.3 (dd, J(C,P) = 14.3, C-3); 125.4 (dd, J(C,P) = 0.6, C-8); 125.2 (sd, J(C,P) = 161.9, C-1); 124.2 (d, C-9); 124.1 (dd, J(C,P) = 10.7, C-5); 121.9 (sd, J(C,P) = 11.5, C-7); 120.0 (dd, J(C,P) = 5.9, C-11); 41.7 (td, J(C,P) = 5.6, C-13).

^{31}P NMR (162.0 MHz, DMSO-d$_6$) δ (ppm): 15.2

^{1}H-^{13}C HMBC: H-2 → C-(1w, 4, 6); H-3 → C-(1, 2w, 5); H-4 → C-(2, 3w, 6); H-5 → C-(1, 3, 7); H-8 → C-(6, 10, 12); H-9 → C-(7, 8w, 11); H-10 → C-(8, 11w, 12); H-11 → C-(7, 9, 12); H-13 → C-(13); NH → C-(13w).

^{1}H-^{1}H DQF-COSY: H-2 → H-(3); H-3 → H-(2, 4); H-4 → H-(3, 5); H-5 → H-(4); H-8 → H-(9); H-9 → H-(8, 10); H-10 → H-(9, 11); H-11 → H-(10); H-13 → H-(14); NH → H-(13).

^{1}H-^{31}P HMBC: H-(2, 3, 5, 13, NH) → P

Minor Isomer (40%)

^{1}H NMR (400.2 MHz, DMSO-d$_{6}$) δ (ppm): 8.14 (m, J(H,P) = 3.1, 2H, H-5); 8.10 (m, 2H, H-8); 7.77 (m, J(H,P) = 22.1, 2H, H-2); 7.70 (m, 2H, H-4); 7.50 (m, J(H,P) = 3, 2H, H-3); 7.39 (m, 2H, H-10); 7.27 (m, 2H, H-9); 7.15 (m, 2H, H-11); 5.75 (m, J(H,P) = 11.8, 2H, NH); 2.85 (m, 4H, H-13).

^{13}C NMR (100.6 MHz, DMSO-d$_{6}$) δ (ppm): 149.3 (sd, J(C,P) = 7.1, C-12); 136.0 (sd, J(C,P) = 6.8, C-6); 132.7 (d, C-4); 130.3 (d, C-10); 129.4 (dd, J(C,P) = 9.7, C-2); 128.3 (dd, J(C,P) = 14.3, C-3); 125.4 (dd, J(C,P) = 0.7, C-8); 125.2 (sd, J(C,P) = 161.9, C-1); 124.2 (d, C-9); 124.1 (dd, J(C,P) = 10.8, C-5); 121.9 (sd, J(C,P) = 11.5, C-7); 120.0 (dd, J(C,P) = 5.6, C-11); 41.7 (td, J(C,P) = 5.6, C-13).

^{31}P NMR (162.0 MHz, DMSO-d$_{6}$) δ (ppm): 15.3

^{1}H-^{13}C HMBC: H-2 → C-(1w, 4, 6); H-3 → C-(1, 2w, 5); H-4 → C-(2, 3w, 6); H-5 → C-(1, 3, 7); H-8 → C-(6, 10, 12); H-9 → C-(7, 8w, 11); H-10 → C-(8, 11w, 12); H-11 → C-(7, 9, 12); H-13 → C-(13); NH → C-(13w).

^{1}H-^{1}H DQF-COSY: H-2 → H-(3); H-3 → H-(2, 4); H-4 → H-(3, 5); H-5 → H-(4); H-8 → H-(9); H-9 → H-(8, 10); H-10 → H-(9, 11); H-11 → H-(10); H-13 → H-(14); NH → H-(13).

^{1}H-^{31}P HMBC: H-(2, 3, 5, 13, NH) → P

ETA-DOPO (2 isomers, ca. 1:1)

^{1}H NMR (400.2 MHz, DMSO-d$_{6}$) δ (ppm): 8.24 (m, J(H,P) = 6.2, 1H, H-18); 8.18 (m, 1H, H-21); 8.14 (m, 1H, H-5); 8.12 (m, 1H, H-8); 7.89 (m, J(H,P) = 14.6, 1H, H-15); 7.83 (m, 1H, H-17); 7.71 (m, 1H, H-4); 7.65 (m, J(H,P) = 14, 1H, H-2); 7.61 (m, J(H,P) = 3.6, 1H, H-16); 7.45 (m, 1H, H-23); 7.44 (m, 1H, H-3); 7.40 (m, 1H, H-10); 7.32 (m, 1H, H-22); 7.28 (m, 1H, H-9); 7.27 (m, 1H, H-24); 7.12 (m, 1H, H-11); 5.83 (m, J(H,P) = 11.8, 1H, NH); 4.02 (m, 2H, H-26); 3.00 (m, 2H, H-13).

^{13}C NMR (100.6 MHz, DMSO-d$_{6}$) δ (ppm): 149.3 (sd, J(C,P) = 7.1, C-12); 149.2 (sd, J(C,P) = 7.9, C-25); 136.2 (sd, J(C,P) = 7.1, C-19); 135.9 (sd, J(C,P) = 7, C-6); 134.0 (dd, J(C,P) = 2.4, C-17); 132.7 (d, C-4); 130.9 (d, C-23); 130.4 (d, C-10); 129.9 (dd, J(C,P) = 9.3, C-15); 129.4 (dd, J(C,P) = 9.7, C-2); 128.7 (dd, J(C,P) = 15.1, C-16); 128.3 (dd, J(C,P) = 14.3, C-3); 125.9 (d, C-21); 125.4 (dd, J(C,P) = 0.6, C-8); 125.3 (sd, J(C,P) = 162.9, C-1); 125.1 (d, C-22); 124.6 (dd, J(C,P) = 11.7, C-18); 124.3 (d, C-9); 124.1 (dd, J(C,P) = 10.7, C-5); 122.0 (sd, J(C,P) = 11.8, C-20); 121.9 (sd, J(C,P) = 11.6, C-7); 121.5 (sd, J(C,P) = 178.9, C-14); 120.1 (dd, J(C,P) = 5.9, C-11); 119.9 (dd, J(C,P) = 6.5, C-24); 66.1 (t, C-26); 40.4 (td, J(C,P) = 7.3, C-13).

^{31}P NMR (162.0 MHz, DMSO-d$_{6}$) δ (ppm): 14.6 (P_a); 9.8 (P_b).

^{1}H-^{13}C HMBC: H-2 → C-(4, 6); H-3 → C-(1, 5); H-4 → C-(2, 6); H-5 → C-(1, 3, 7); H-8 → C-(6, 10, 12); H-9 → C-(7, 11); H-10 → C-(8, 12); H-11 → C-(7, 9, 12); H-13 → C-(26); NH → H-(13); H-15 → C-(17, 19); H-16 → C-(14, 18); H-17 → C-(15, 19); H-18 → C-(6, 14, 20); H-21 → C-(19, 23, 25); H-22 → C-(20, 24); H-23 → C-(21, 25); H-24 → C-(20, 22, 25); H-26 → C-(13).

^{1}H-^{1}H DQF-COSY: H-2 → H-(3); H-3 → H-(2, 4); H-4 → H-(3, 5); H-5 → H-(4); H-8 → H-(9); H-9 → H-(8, 10); H-10 → H-(9, 11); H-11 → H-(10); H-13 → H-(NH, 26); H-15 → H-(16); H-16 → H-(15, 17); H-17 → H-(16, 18); H-18 → H-(17); H-21 → H-(22); H-22 → H-(21, 23); H-23 → H-(22, 24); H-24 → H-(23); H-26 → H-(13).

^{1}H-^{31}P HMBC: H-(2, 3, 5, 13, NH) → P_a; H-(15, 16, 18, 26) → P_b

Figure S1a. ^{1}H, ^{13}C and ^{31}P NMR spectra of EG-DOPO (DMSO-d_6).

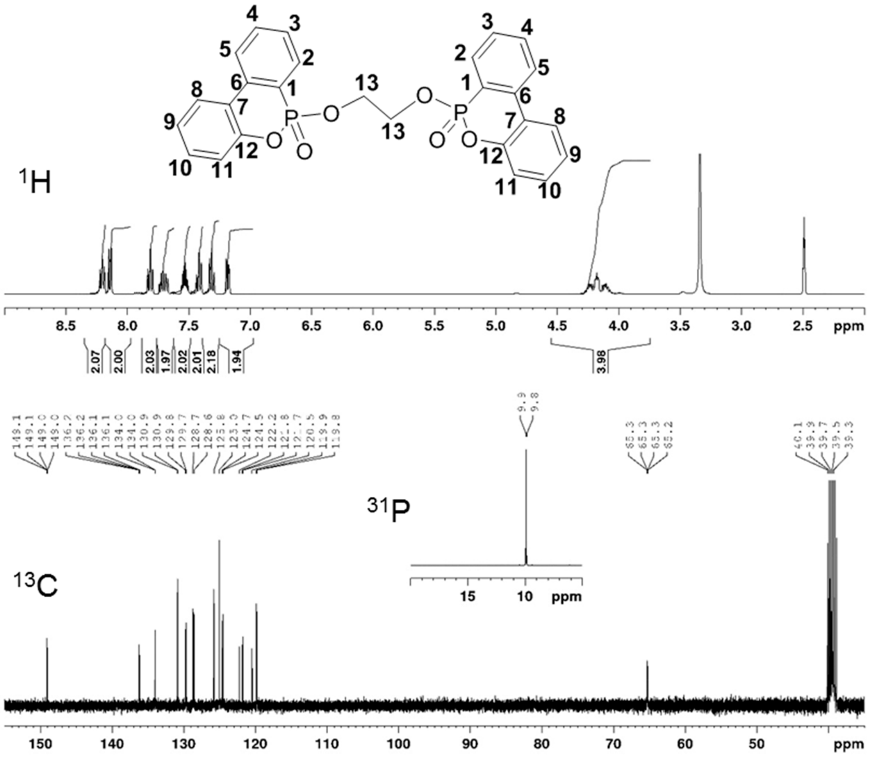

Figure S1b. Regions of interest of ^{1}H-^{13}C HSQC (**A**, **B**), ^{1}H-^{13}C HMBC (**C**), and ^{1}H-^{1}H DQF-COSY (**D**) NMR spectra of EG-DOPO (DMSO-d_6).

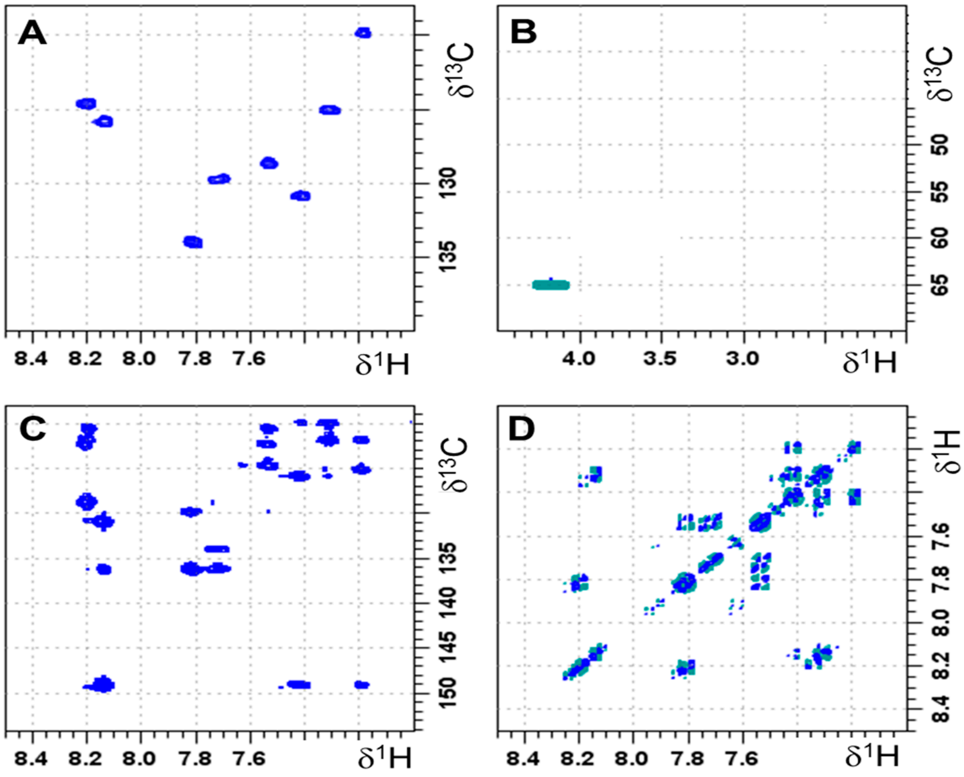

Figure S1c. ^{1}H, ^{13}C and ^{31}P NMR spectra of ETA-DOPO (DMSO-d$_6$)

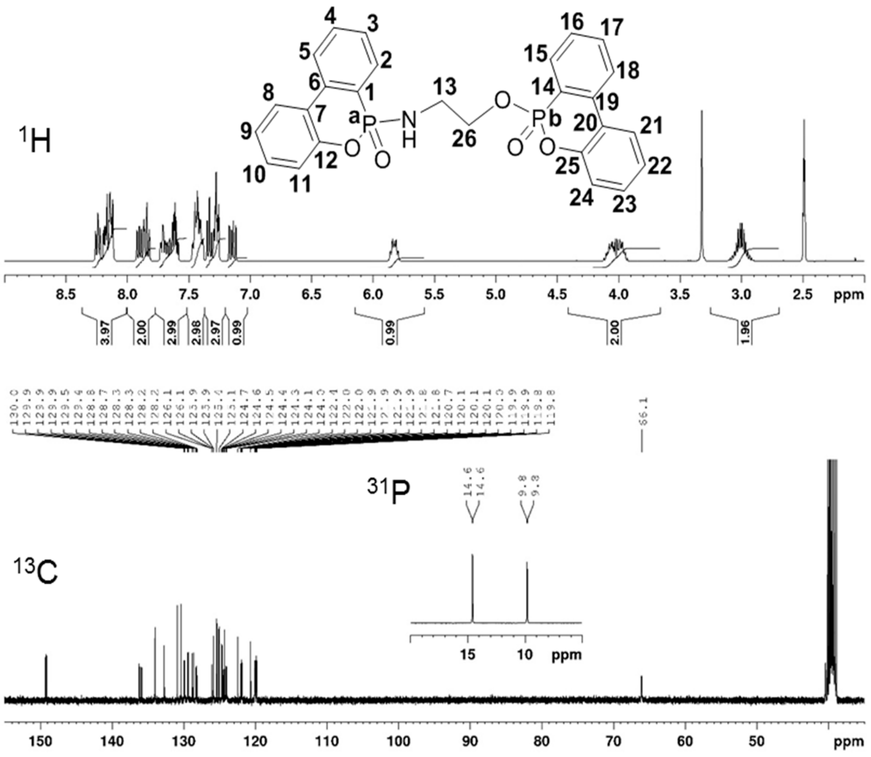

Figure S1d. Regions of interest of ^{1}H-^{13}C HSQC (**A**, **B**), ^{1}H-^{13}C HMBC (**C**) and ^{1}H-^{1}H DQF-COSY (**D**) NMR spectra of ETA-DOPO (DMSO-d$_6$).

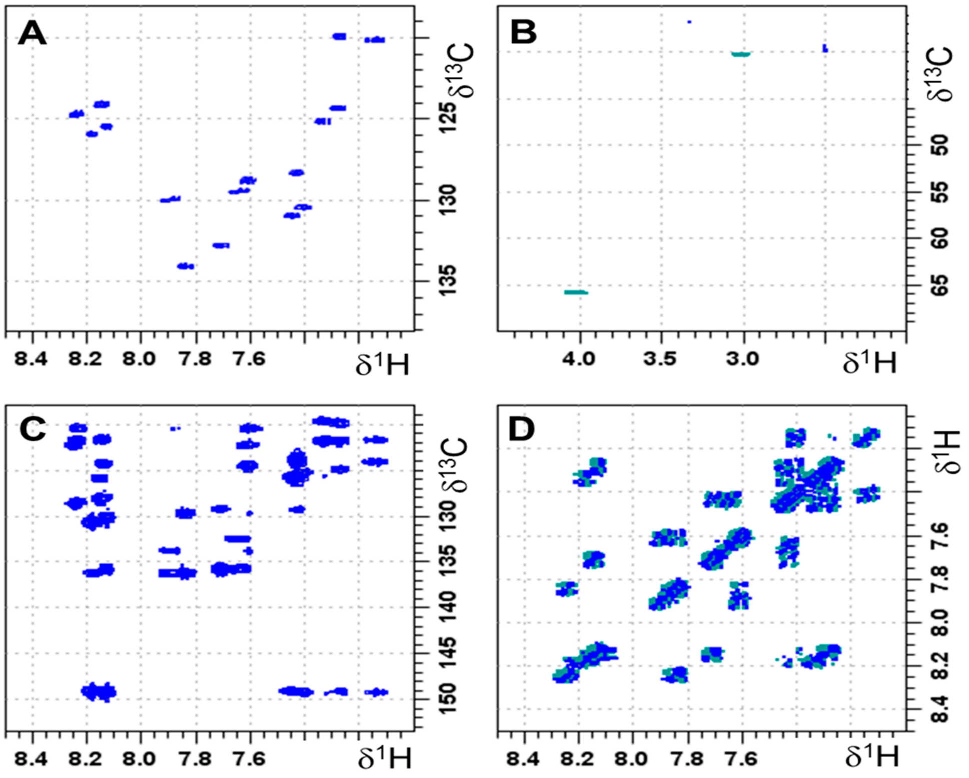

Figure S1e. ^{1}H, ^{13}C, and ^{31}P NMR spectra of EDA-DOPO (DMSO-d_6).

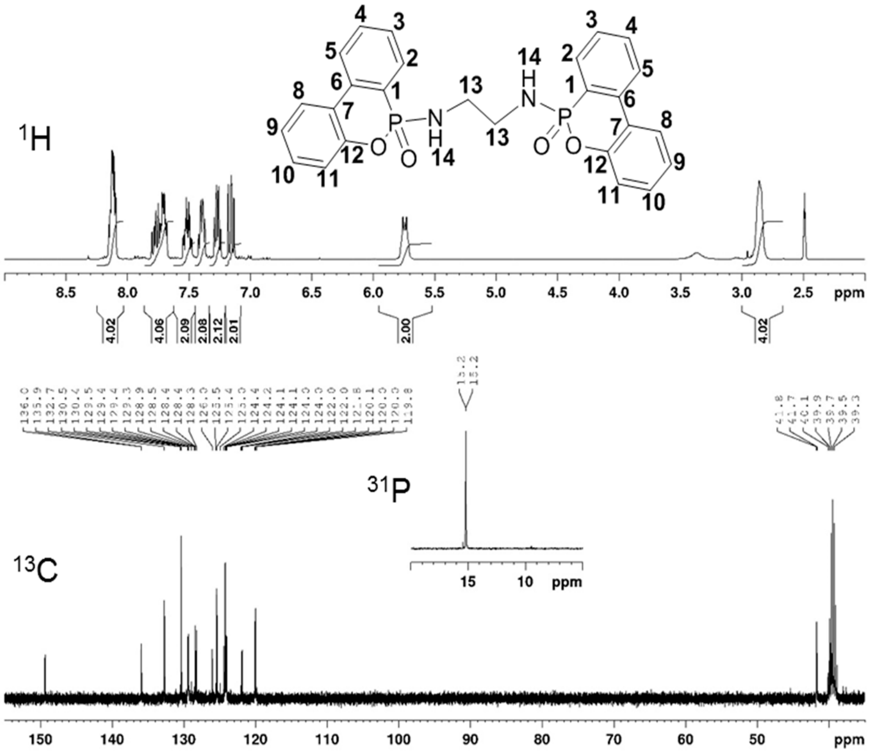

Figure S1f. Regions of interest of ^{1}H-^{13}C HSQC (**A**, **B**), ^{1}H-^{13}C HMBC (**C**), and ^{1}H-^{1}H DQF-COSY (**D**) NMR spectra of EDA-DOPO (DMSO-d_6),

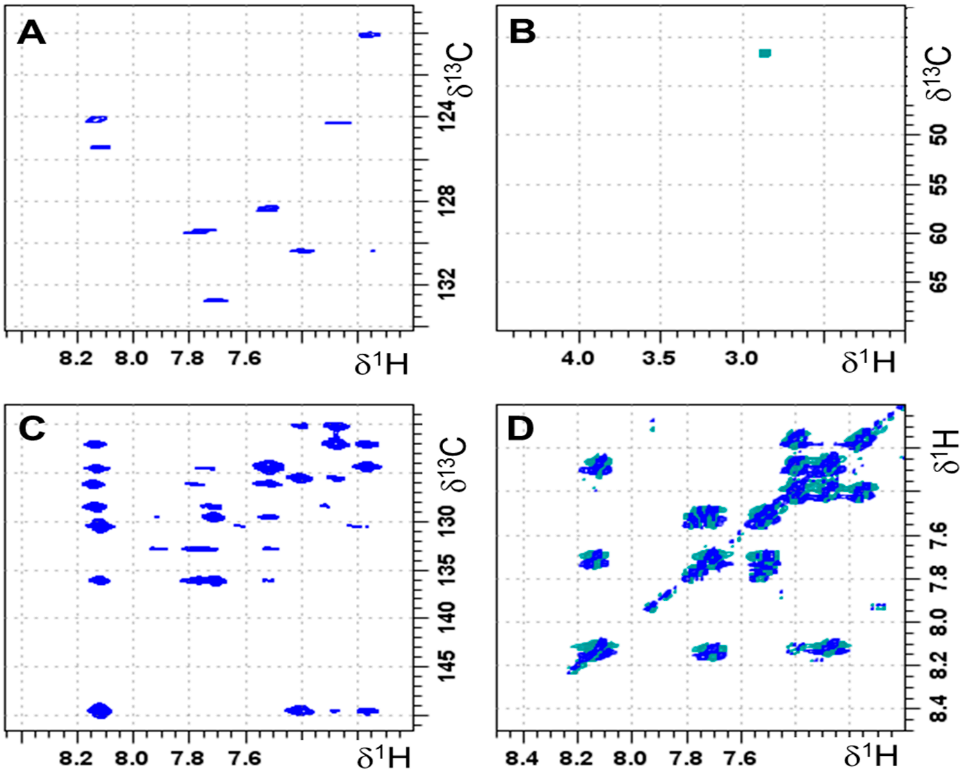

Figure S2. TGA data of bridged DOPO compounds (N_2 atmosphere).

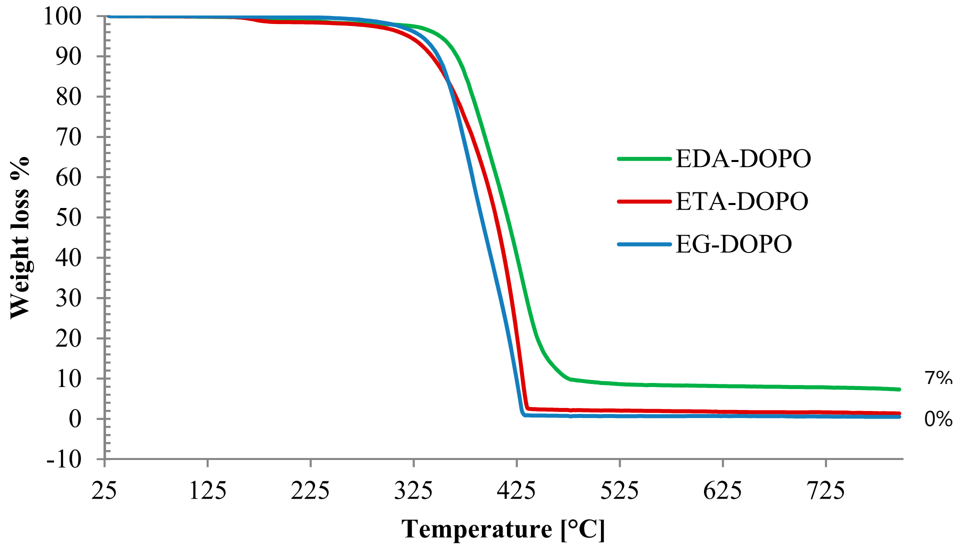

Figure S3. TGA data of PU foams containing 5% bridged DOPO compounds (N_2 atmosphere).

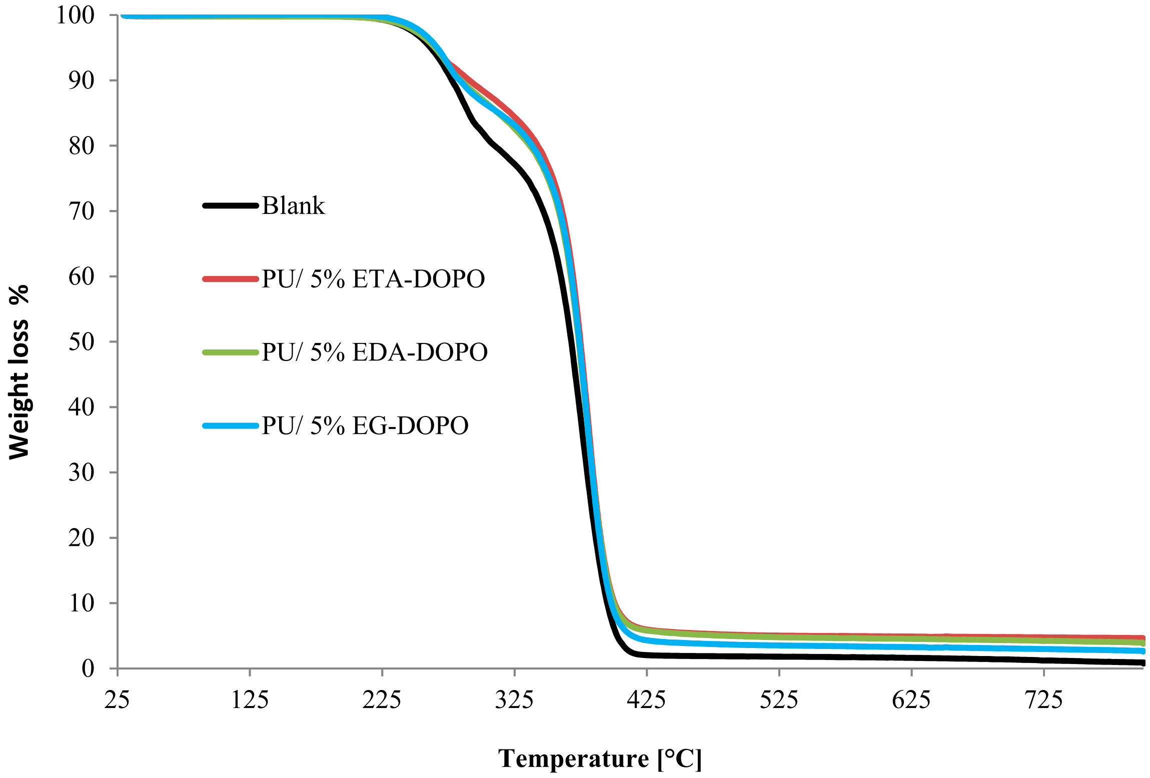

Supplement: Supplementary file 1 [file polymers-10-01115-s001.pdf]
